# Problematic internet use among elementary school students during the Covid – 19 pandemic

**DOI:** 10.1192/j.eurpsy.2022.501

**Published:** 2022-09-01

**Authors:** L. Iliopoulou, V. Koutras

**Affiliations:** 1 General Hospitai of Ioannina, Phyciatric, Ioannina, Greece; 2 University of Ioannina, Early Childhood Education, Ioannina, Greece

**Keywords:** Covid-19, elementery students, internet, problematic use

## Abstract

**Introduction:**

During Covid-19 pandemic schools in Greece were closed and distance education instituted.

**Objectives:**

To find out whether the pandemic circumstances and the catholic internet access affected the time students spend on web activities other than educational duties.

**Methods:**

Our sample consisted of 1213 parents with children from 4th, 5th, and 6th elementary school grades. They were collected with snowball sampling through internet, and they filled closed ended questions anonymous questionary.

**Results:**

During the pandemic the amount of time that children spent on the internet for purposes other than school obligations (social media, videogames, videos) was increasing by the time. On holidays and weekends this time was further increased. Parents mentioned reduced sleep time, reduced interest in hobbies and activities, as well as in person social communication with friends and loss of interest for school and educational matters. Children spend a lot of time on internet activities and sometimes they use it to avoid loneliness and negative situations. According to parents if their child doesn’t spend its preferrable time on the internet, get anxious, irritable, and sad. Often the child hides the time of internet use. Children from families with low socioeconomic, educational level, family income are more vulnerable to develop internet problematic use.

**Conclusions:**

Social isolation, school closures, distance education, cancellation of after school activities and the facile internet access increased problematic internet use. This use is associated with behavioural, emotional and psychosocial problems. It is important to give information and implement educational programs for parents about how to control internet use of their children.

**Disclosure:**

No significant relationships.

